# Does the initiation of urate-lowering treatment during an acute gout attack prolong the current episode and precipitate recurrent attacks: a systematic literature review

**DOI:** 10.1007/s00296-016-3579-z

**Published:** 2016-10-19

**Authors:** Fatma Eminaga, Jonathan Le-Carratt, Adrian Jones, A. Abhishek

**Affiliations:** 1 Department of Medicine, Nottingham University Hospitals NHS Trust, Nottingham, NG7 2UH UK; 2 Academic Rheumatology, Clinical Sciences Building, University of Nottingham, Nottingham, NG5 1PB UK

**Keywords:** Acute gout, Urate-lowering treatment, Systematic review

## Abstract

The aim of this study was to systematically review the literature on effect of initiating urate-lowering treatment (ULT) during an acute attack of gout on duration of index attack and persistence on ULT. OVID (Medline), EMBASE and AMED were searched to identify randomized controlled trials (RCTs) of ULT initiation during acute gout attack published in English language. Two reviewers appraised the study quality and extracted data independently. Standardized mean difference (SMD) and relative risk (RR) were used to pool continuous and categorical data. Meta-analysis was carried out using STATA version 14. A total of 537 studies were selected. A total of 487 titles and abstracts were reviewed after removing duplicates. Three RCTs were identified. There was evidence from two high-quality studies that early initiation of allopurinol did not increase pain severity at days 10–15 [SMD_pooled_ (95 % CI) 0.18 (−0.58, 0.93)]. Data from three studies suggested that initiation of ULT during an acute attack of gout did not associate with dropouts [RR_pooled_ (95 % CI) 1.16 (0.58, 2.31)]. There is moderate-quality evidence that the initiation of ULT during an acute attack of gout does not increase pain severity and risk of ULT discontinuation. Larger studies are required to confirm these findings so that patients with acute gout can be initiated on ULT with confidence.

**Electronic supplementary material:**

The online version of this article (doi:10.1007/s00296-016-3579-z) contains supplementary material, which is available to authorized users.

## Introduction

Gout is the commonest inflammatory arthritis and results from monosodium urate (MSU) crystal deposition. It is the only arthritis that has the potential of being cured if the serum uric acid is reduced to <360 µmol/L persistently with effective urate-lowering treatment (ULT) [1]. Guidelines for the management of gout recommend that patients continue on ULT during a gout attack, but provide conflicting recommendations on whether ULT can be started during an acute attack [1–3]. For example, while the British Society for Rheumatology (BSR) and European League Against Rheumatism (EULAR) guidelines suggest commencing ULT 1–2 weeks after the acute attack has resolved, the 2012 American College of Rheumatology (ACR) guidelines suggest that ULT may be started during an acute attack [1–3]. However, these recommendations are discordant, and the latest Cochrane review did not examine the effect of ULT initiation during an acute attack of gout on its duration and severity [4]. Thus, further research is required to examine whether ULT can be initiated during an acute gout attack, without unduly prolonging the index episode.

The objectives of this study were to systematically review the literature to identify studies examining the effect of initiation of ULT during an acute gout attack on severity of the index episode, recurrent gout attacks, persistence on ULT and to conduct a meta-analysis to provide estimate of the effects.

## Methods

### Literature search

MeSH terms were used to identify randomized controlled trials (RCTs) of ULT for gout in OVID (Medline) R 1946–week 4 October 2015, EMBASE (1974–week 44, 2015) and AMED (1985–October 2015). Reference lists of eligible studies, ACR, EULAR, BSR guidelines and Cochrane systematic reviews of pharmacologic treatment of gout were hand searched. We restricted our search to studies in English. PubMed was searched using free text terms combining each ULT (allopurinol, febuxostat, benzbromarone and probenecid, etc.) and “acute gout” to identify any additional studies.

### Inclusion criteria

Initiation of any ULT in patients with acute gout (either new diagnosis or long-standing gout) and ≥18 years in age.

### Exclusion criteria

Up-titration of ULT dose during an acute attack, ULT initiation in the intercritical period, animal studies and conference abstracts.

### Study selection

The titles and abstracts of retrieved studies were independently reviewed by two reviewers. Full text articles were obtained if more information was required to decide eligibility.

### Quality assessment

Two independent reviewers appraised the selected studies for quality. Studies were evaluated using the Cochrane assessing risk of bias tool and Jadad score. Any disagreement was resolved after discussion with independent reviewer. The quality of evidence was assessed using the Cochrane collaborations’ GRADE tool.

### Data extraction

Data extraction was performed independently by two reviewers. Discrepancies were resolved after discussion with an independent reviewer. For each study, reviewers extracted data that were deemed to potentially impact efficacy outcomes, such as study population (percent men, mean age, disease duration, serum uric acid), study design (dose of ULT, initial treatment of gout attack, gout attack prophylaxis) and outcomes (total duration of gout attack, recurrent gout attacks, pain severity, ULT discontinuation). For continuous outcomes, data were pooled with the standardized mean difference (SMD) of the final value across groups. For dichotomous data, the relative risk (RR) and 95 % confidence interval (CI) were calculated. Meta-analysis was performed using the random effect model. *I* ^2^ was calculated to assess heterogeneity. Meta-analysis was carried out using STATA version 14.

## Results

**Fig. 1:**
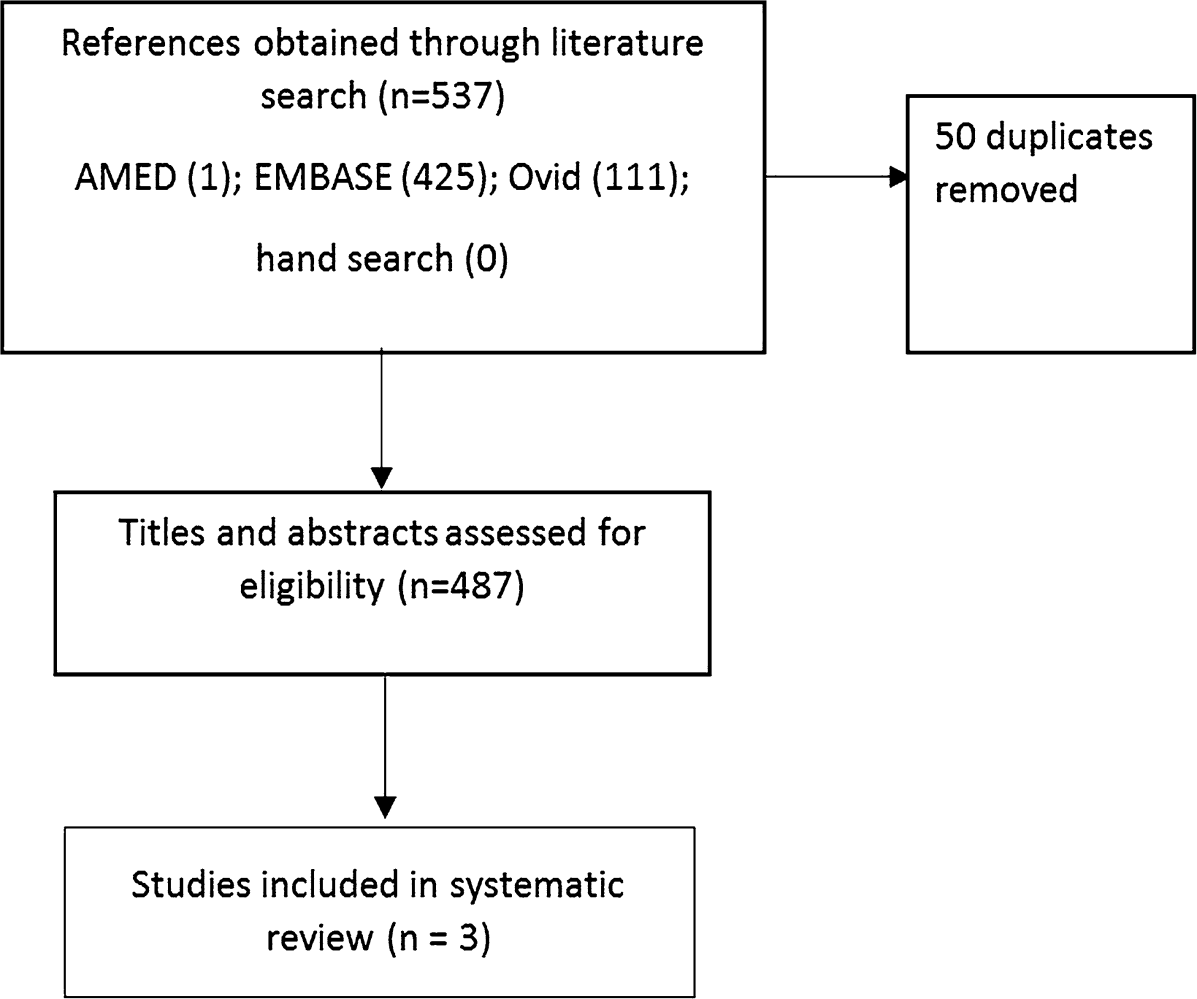
Flowchart summarizing study selection

Three studies were eligible to be included in this systematic review (Fig. 1) [5–7]. Their details are in Table S1. Two of these studies were of high quality according to the Cochrane assessing risk of bias tool (Table 1). Data from only the first 28 days from one of the studies could be included [5] as that study compared the effect of azapropazone (a uricosuric anti-inflammatory drug) or regular indomethacin followed by delayed initiation of allopurinol on day 28, on treatment of the acute attack of gout and in preventing recurrent gout attacks [5]. Patients initially randomized to indomethacin were required to discontinue indomethacin and start allopurinol on day 28, which resulted in only part of the data being eligible for included in this systematic review.

**Table 1:** Quality of included studies according to the risk of bias assessment tool and Jadad score

	Random sequence generation (selection bias)	Allocation concealment (selection bias)	Blinding of participants and personnel (performance bias)	Blinding of outcome assessment (detection bias)	Incomplete outcome data (attrition bias)	Selective reporting (reporting bias)	Other biases	Jadad score	Quality of evidence (GRADE scheme)
Hill	+	+	+	+	−	−	−/−	5/5	M
Taylor	+	+	+	+	−	−	−/−	5/5	M
Fraser	?	?	+	+	−	−	−/−	4/5	M

### Duration of gout attack

Information on duration of gout attack was only provided in one study and did not differ significantly between the allopurinol and placebo arms [17 (8.53) vs. 12.53 (7.73) days], *p* = 0.13 (intention to treat analysis) [7]. Authors of other studies were contacted, but were unable to provide any additional unpublished data on the duration of gout attack.

### Pain visual analogue score (VAS) by day 10

**Fig. 2:**
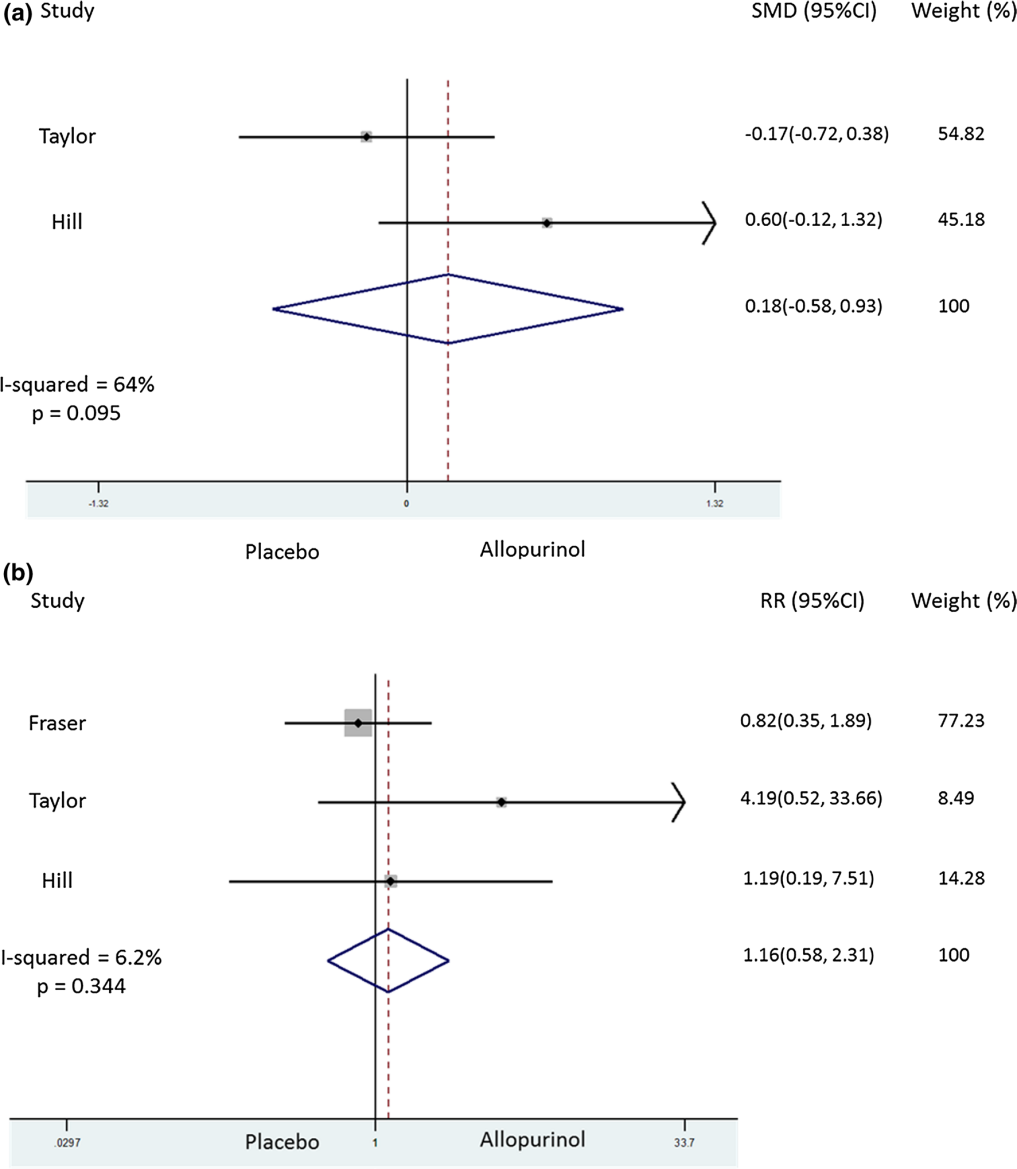
Forest plot showing effect of early initiation of ULT on **a** pain at day 10 (*top panel*) and **b** dropouts (*lower panel*)

Information on pain VAS on day 10 was published in one paper [6] and was obtained by contacting the authors of another study [7]. In the latter study, pain VAS was measured between days 10 and 15 [7]. On pooling the data, there was no evidence to suggest that early initiation of ULT associated with pain; however, there was substantial heterogeneity, with *I* ^2^ = 64 % (Fig. 2a). There was no evidence of a publication bias (Figure S1).

### Recurrent gout attacks

The comparison groups and end points were heterogeneous and did not allow for pooling of data on recurrent gout attacks. In the study comparing early versus delayed initiation of allopurinol, the number of patients with acute gout attacks was comparable in the early (7.7 %) and delayed (12 %) initiation arms at 28 days [6]. Only 1 patient commenced on allopurinol had a flare of acute gout in the Hill et al.’s [7] study; however, the other group was only given placebo, which would not be expected to trigger acute gout. In the study by Fraser et al. [5], there was one acute attack of gout in the first 28 days in those randomized to azapropazone, while there were 5 acute attacks of gout in those randomized to indomethacin.

### Dropouts/medication adherence

Information on dropouts by day 28 [5, 6]–30 [7] was available for all three studies. Hill et al. [7] reported that the dropout rate was comparable by day 10 (1/16 allopurinol vs. 1/19 placebo). Meta-analysis of included studies showed that early ULT initiation did not associate with excessive dropouts (Fig. 2b). However, although the number of studies was limited, there seemed to be publication bias for this outcome (Figure S1).

### Adverse events

The adverse event rates were comparable in those commenced on ULT during an acute attack of gout (Table S2).

### Inflammation markers

Data on C-reactive protein and erythrocyte sedimentation rates in the follow-up period were only reported in one study and demonstrated a nonsignificant reduction in CRP and ESR at days 10 and 30 in those initiated on allopurinol during an acute attack of gout [6].

## Discussion

This systematic review collected evidence from all RCTs of ULT initiation during acute gout attack published in the English language. We retrieved 3 trials, including 185 gout patients initiated on allopurinol [6, 7] or azapropazone [5] during a gout attack. Two trials were at low [6, 7] and one [5] at unclear risk of bias. However, the studies provide moderate-quality evidence due to small sample size [6, 7] or lack of suitable control group [5].

Our findings imply that ULT initiated during an acute gout attack does not prolong the index attack. However, there was significant heterogeneity in the findings of the included studies [6, 7]. This may be due to the fact that the treatment of acute gout was not standardized in one study [7], and the disease characteristics of gout patients in the studies by Taylor et al. and Hill et al. were different. For example, patients in the study by Taylor et al. had milder disease (more likely to be presenting with gout for the first time) and did not have tophaceous gout, while those in the study by Hill et al. frequently had tophaceous gout [6, 7]. This suggests that the initiation of ULT during an acute attack of gout may be safer in patients with early stages of gout than in those with advanced tophaceous gout.

Apart from this, dropout rates in the early ULT initiation group and placebo or delayed ULT initiation groups were comparable, suggesting that ULT initiated during an acute gout attack is well tolerated. However, although not statistically significant, the dropout rates in early ULT initiation group were higher in one study in which allopurinol was started at 300 mg/day [6], compared to the study in which allopurinol was initiated at 100 mg/day as per the ACR and EULAR recommendations [7], suggesting that ULT should be initiated at a low dose and up-titrated gradually. The difference in dropout rates between the two studies is not likely to be due to treatment of acute attack of gout, as both NSAIDs and corticosteroids are equally effective in treating acute attacks [8].

A hospital-based retrospective study which examined the effect of initiation of ULT during an acute gout attack reported that patients commenced on ULT during an acute attack were significantly more likely to meet the SUA target earlier and less likely to have chronic kidney disease in the long term [9]. However, the findings of this study may be biased due to its retrospective observational nature.

Initiation of ULT during a gout attack can increase ULT prescription rates. This is as symptoms of gout are initially intermittent, and many patients do not return for ULT after the acute attack has resolved. Thus, early initiation of ULT can improve overall gout treatment and reduce healthcare cost by avoiding further visits to initiate ULT.

In summary, ULT initiated during an acute gout attack is well tolerated and does not prolong the index episode. However, there are several caveats to this study, namely, small sample size of included studies, heterogeneous study population and nonstandardized treatment of acute gout in one study and allopurinol initiation at a high dose in another study. Similarly, in the absence of individual patient data, and the fact that two of the three studies included in this systematic review did not report data on inflammatory markers during the follow-up period, we are unable to examine the relationship between (a) reduction in serum uric acid levels and changes in pain VAS and (b) early initiation of ULT and reduction in inflammation. Thus, further adequately powered studies that examine the effect of initiating slow up-titrated ULT during an acute attack of gout on index attack duration and severity are needed before this strategy of ULT initiation can be widely implemented.

## Electronic supplementary material

Below is the link to the electronic supplementary material.

Supplementary material 1 (DOCX 35 kb)
