# Supplementary material for: Does the initiation of urate-lowering treatment during an acute gout attack prolong the current episode and precipitate recurrent attacks: a systematic literature review
Source: Rheumatol Int. 2016 Oct 19;36(12):1747–52. doi: 10.1007/s00296-016-3579-z (PMC5102948; doi:10.1007/s00296-016-3579-z)
Supplement: Supplementary file 1 — Supplementary material 1 (DOCX 35 kb) [file 296_2016_3579_MOESM1_ESM.docx]

Table S1: Baseline characteristics of included studies

|  | Fraser, 1987 | Taylor, 2012 | Hill, 2015 |
| --- | --- | --- | --- |
| Study design | RCT | RCT | RCT |
| Setting | Academic primary care + hospital clinic | Community clinic (Veteran’s Administration) | USA Air Force Hospital |
| Country | UK | USA | USA |
| Sample Size A/C | 46/47 | 31/26 | 16/19 |
| Age, Mean A/C | Not reported^1^ | 57/61 | 60.63/53.11 |
| Male A/C | Not reported^1^ | 31/26 | 16/17 |
| BMI, Mean A/C | Not reported | 32/32 | Not reported |
| Disease duration A/C | Not reported | 4.1/6.5 | 5.5 / 4.9 |
| Serum Uric acid at baseline, Mean, A/C | Not reported | 7.8/7.6 | 8.16/7.95 |
| % with tophi A/C | Not reported | 0%/0% | 37.5%/31.5% |
| % monoarticular acute gout A/C | Not reported | 100%/88% | 62%/58% |
| Treatment for acute gout at recruitment | Azapropazone^2^ or indomethacin | Indomethacin for 10 days to treat the acute attack | 81.3% received p/o, i/a, i/m corticosteroids (12.5% in the allopurinol vs. 42.1% in the placebo arm received i/a corticosteroids, p=0.07) |
| Duration of treatment | 85 days blinded: open label extension to 225 days | 10 days: both groups commended on allopurinol open label at day 11 and continued to day 30 | 28 days |
| Intervention active group | Azapropazone^2^  1.8 gm/day days1-28,  1.2 gm/day days 29-225 | Allopurinol 300mg/day | Allopurinol, initiated an up-titrated as per ACR recommendations |
| Intervention control group | Indomethacin days1-28, allopurinol days 29-225 | Placebo | Placebo |
| Prophylaxis | Azapropazone^1^ (A) or indomethacin (C) | Colchicine | Colchicine all (1 meloxicam, 1 no prophylaxis) |

^1^Similar age and sex distribution in active and control groups; ^2^Azapropazone is a uricosuric drug with anti-inflammatory effects.

Table S2 : Summary of adverse events

|  | Fraser, 1987^1^ | Taylor, 2012 | Hill, 2015 |
| --- | --- | --- | --- |
| SAE including death | Active - Perforated peptic ulcer (1)  Control - GI bleed (1) | Active – multi-organ failure  (1, died)  Control- allopurinol hypersensitivity syndrome (1) | None |
| Other adverse events | Active – GI (11), rash (1) Control – GI (6), rash (1), CNS (3), CCF (1), Liver (1) | Active – elevated creatinine (1)  Control- elevated creatinine (1) | Active - epistaxis on warfarin (1); Placebo - nausea & vomiting (1), deranged LFTs (1) |


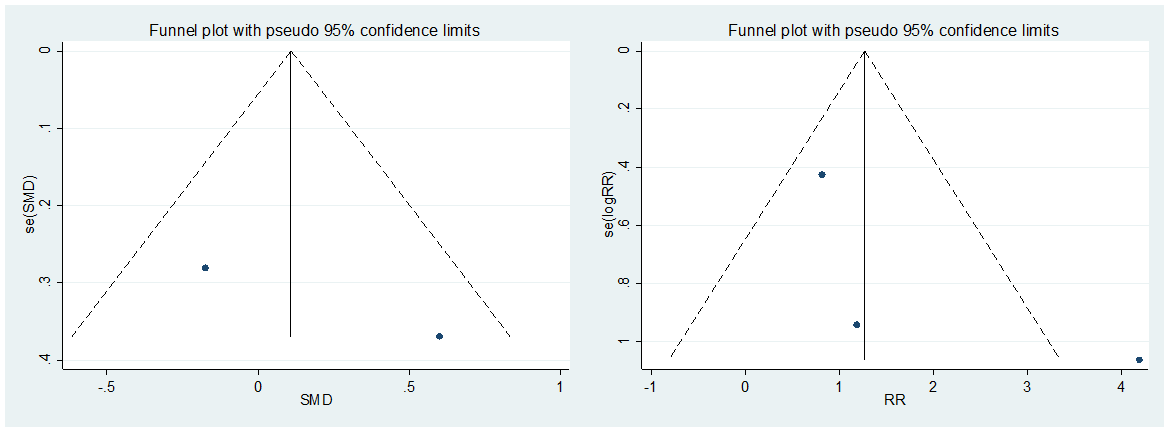


**Figure S1** Funnel plot demonstrating publication bias in pain visual analogue score by day 10 (left panel) and publication bias in dropouts (right panel).
